# Comparison of Three Histological Techniques for Fat Emboli Detection in Lung Cetacean’s Tissue

**DOI:** 10.1038/s41598-020-64821-8

**Published:** 2020-05-19

**Authors:** Marina Arregui, Antonio Fernández, Yania Paz-Sánchez, Ángelo Santana, Simona Sacchini, Eva Sierra, Manuel Arbelo, Yara Bernaldo de Quirós

**Affiliations:** 1 Atlantic Cetacean Research Center, Institute of Animal Health (IUSA), Veterinary School, University of Las Palmas de Gran Canaria (ULPGC), Trasmontaña, s/n, 35413 Arucas, Las Palmas Spain; 2 Department of Mathematics, University of Las Palmas de Gran Canaria (ULPGC), Campus de Tafira s/n, 35017 Las Palmas, Spain

**Keywords:** Microscopy, Trauma

## Abstract

Fat embolism is the mechanical blockage of blood vessels by circulating fat particles. It is frequently related to traumas involving soft tissues and fat-containing bones. Different techniques have been used for decades to demonstrate histologically fat emboli, being the extremely toxic post-fixation with osmium tetroxide one of the most used techniques in the last decades. In the present study, the osmium tetroxide technique was compared qualitatively and quantitatively, for the first time, with chromic acid and Oil Red O frozen techniques  for histological fat emboli detection in the lungs of eight sperm whales that died due to ship strikes. This was also the first time that chromic acid technique was tested in cetaceans. Results showed that the three techniques were valuable for the histological detection of fat embolism in cetaceans, even when tissues presented advanced autolysis and had been stored in formaldehyde for years. Although quantitative differences could not be established, the Oil Red O frozen technique showed the lowest quality for fat emboli staining. On the contrary, the chromic acid technique was proven to be a good alternative to osmium tetroxide due to its slightly lower toxicity, its equivalent or even superior capacity of fat emboli detection, and its significantly lower economic cost.

## Introduction

Fat embolism is defined as the mechanical obstruction of blood vessels by circulating fat particles^1,2^. The lung has traditionally been considered the target organ for fat emboli detection, as the pulmonary microvasculature (<20 µm in diameter) blocks the circulation of the fat droplets, acting as a filter to protect the systemic circulation^2,3^.

Different techniques have been used for histological fat emboli demonstration, usually under light microscopy or less frequently using electron^4,5^ or most recently, confocal microscopy^6,7^. While the electron and confocal microscopy techniques are very interesting and promising technologies for the investigation on different aspects of fat emboli, they require expensive equipment and trained personnel thus, they are not accessible to many diagnostic laboratories. At the same time, the more classic histopathological techniques are routinely done by most laboratories and can provide a proper diagnosis. Considering this, the present study will be focused on histological fat embolism diagnosis using light microscopy.

Among the various techniques used for lipid demonstration in frozen sections of fresh or formalin-fixed tissues, the solvent dyes Sudan III and IV^8,9^ were the first to be used (e.g.^10–12^). Later, other solvent dyes as Sudan Black B or Oil Red O, argued to give superior results^13,14^, started to be commonly used^1,15–17^. Frozen techniques for lipid staining comprise lower health risks but have been argued to give lower morphology quality and to show less capacity to detect small quantities of lipids in comparison to techniques in paraffin-embedded tissues^18,19^.

In paraffin-embedded tissues, osmium tetroxide (OsO_4_) has been for decades the technique of choice for histological demonstration of fat embolism due to its high quality^20–24^. As lipids are soluble in the processing solvents used to embed the tissues in paraffin, formalin-fixed tissues should be treated with OsO_4_ to fix the lipids to the sample before paraffin embedding. Nevertheless, when working with OsO_4_ safety measures should be strictly followed as it is known to be extremely toxic. Its vapors can lead to severe eye irritation, including cornea damage, and it is listed by the Globally Harmonized System of Classification and Labeling of Chemicals (GHS)^25^ as fatal if inhaled, swallowed or in contact with skin^26^. In the last decades, Tracy & Walia (2002) have described another post-fixation technique that uses chromic acid to fix lipids by transforming fat emboli in organometallic complexes insoluble in organic solvents and stainable in paraffin sections^4^. Some advantages of chromic acid when compared with OsO_4_ may include its lower price, higher capacity of tissue penetration, and its lower degree in toxicity in some GHS hazard statements. However, chromic acid is a very hazardous and possibly carcinogenic chemical that, as OsO_4_, should be handled carefully and safely^27^.

Fat embolism has been extensively described, both in human and animal science, associated with a variety of traumatic and non-traumatic processes^28,29^, comprising the majority of the cases traumas to soft tissues and fat-containing bones, especially long bones and pelvis^2,30^. Moreover, the histologic detection of lung fat emboli has been used for decades in forensic practice to demonstrate *antemortem* violence, as cardiac function is needed, even for a short time before death, to allow the circulation of fat emboli to the lungs^10,31–33^.

The diagnosis of fat embolism is based on the histological demonstration of fat emboli within vascular vessels. However, not only their presence or absence within the tissue section should be assessed but also graded on a scale. This is necessary because the higher the severity, the greater the probability that fat embolism is involved in the patient´s death. Although there is not a universally accepted system to grade fat embolism, previous studies in the literature have traditionally graded it using semi-quantitative indexes, which are based on the number of emboli encountered in the tissue section analyzed, like Falzi, Sevitt or Mason’s scales^31,34,35^. Some limitations of these semi-quantitative grading systems include that they are based on the observer’s personal criteria, that they do not take into account the size of the emboli (which is expected to play a role in severity), and finally, that they do not consider the total tissue section area, which has been mentioned by previous authors as important to accurately assess the degree of fat embolization^6,36^.

In addition to the need of using objective quantitative scales rather than semi-quantitative indexes to properly assess the morphological severity of fat embolism, it is important to determine if the most commonly used techniques for fat embolism demonstration are equally efficient in the detection and quantification of those emboli, with the objective of using the less toxic but efficient technique. However, to the best of our knowledge there is only one study in the literature that has compared two techniques, the OsO_4_ and Oil Red O frozen section techniques, for their capacity to detect fat emboli but did it in an eye-based study rather than a proper quantitative comparison^18^.

Contrary to the high number of studies in literature addressing fat embolism in different species, especially humans, studies on fat embolism in cetaceans are extremely scarce. This is explained because the field of pathology and histopathology in these animals constitutes a recent research field. Moreover, laboratory techniques and protocols need to be adequate for these species’ tissues.

In cetaceans, fat embolism has been described due to intra-interspecific traumatic interactions^37^, in combination with gas embolism (gas and fat embolic syndrome) in beaked whales that stranded in temporal and spatial association with naval military manouvres^38,39^, and in animals that have been struck by vessels in the Canary Islands^37,40–42^. In this last case of ship strikes, lung fat emboli have proven to be a confirmatory diagnostic tool of *antemortem* strikes, especially in decomposed carcasses in which the assessment of tissular changes characteristic of an *antemortem* trauma can be challenging. In relation to this, the study carried out by Arregui *et al*. in 2019, focused on sperm whales and ships, demonstrated that at least 80% of the specimens with evidence of ship-strike in this area were alive at the moment of the strike and that mitigation measures need to be implemented^42^.

Considering all this, the objectives of the present study were first, to evaluate the capacity of chromic acid to demonstrate fat emboli in cetacean tissues; and secondly, to compare qualitatively (e.g. intensity of the staining or the precise location of fat emboli within vessels) and quantitatively (to assess if they detect a similar quantity of fat emboli) the capacity of fat emboli detection between OsO_4_ to both: the Oil Red O frozen and the chromic acid techniques.

To the best of our knowledge, this is the first study evaluating the chromic acid technique in cetacean lungs positive to fat embolism and the first time a detailed quantitative and qualitative comparison is made among the three techniques for fat emboli detection.

## Material and Methods

### Animals included in the study

To assess and compare the ability of the three histochemistry techniques (i.e.: chromic acid and OsO_4_ in paraffin tissues and Oil Red O in frozen tissues) to stain fat emboli, we selected eight sperm whales, in different conservation codes, which had signs of ship strikes and were OsO_4_-positive to lung fat emboli with different degree of severity^42^.

Lung samples were collected following standardized necropsies^43^. For decomposition status, five codes were applied^43^: code 1 for “very fresh”, was assigned to an animal that has recently died, code 2 for “fresh dead animals” (no bloating nor changes in coloration, eatable meat), code 3 for “moderate autolysis” (may present some skin desquamation, the carcass might have started to swollen, and organs may have changed coloration and be more friable), code 4 for “advanced autolysis” (skin desquamation, swollen carcass, organs difficult to recognize), and code 5 for “very advanced autolysis” (the skin may be absent, some or all organs may be liquefied, and mummification or adipocere may be observed in some carcasses). Among the eight animals included, there was one fresh animal (code 2), one moderately decomposed animal (code 3), five advanced-autolytic animals (code 4), and one very advanced-autolytic animal (code 5). The bias of the study towards decomposed animals was explained because most of the sperm whales that appear floating or stranded in Canarian coasts presented and advanced decomposition state^37,40^.

### Description of the techniques used for fat emboli detection

After collection, lung samples were immediately immersed in 10% neutral buffered formalin for fixation and stored in the Institute of Animal Health Tissue Bank until being processed for the present study. They were treated for fat emboli detection following three techniques, two of them in paraffin-embedded tissues (“OsO_4_ and chromic acid techniques”) and the third one using frozen sections (“frozen section technique”).

As lipids are soluble in the solvents used to embed the tissues in paraffin, OsO_4_ or chromic acid were used in two of the techniques to fix the lipids to the tissue prior to paraffin embedding. In the frozen section technique, lipid fixation was not needed as tissues were not embedded in paraffin.

To compare the OsO_4_ technique with both, the chromic acid- and the frozen section technique, formalin-fixed lung sections were cut in half, being one of the faces treated following the chromic acid technique, and the other one with the OsO_4_ technique. Then, the same or a different lung area was cut in half and one of the faces was treated with OsO_4_ and the other with the frozen section technique, in order to compare both.

Sections of blubber, a specialized hypodermis, and *rete mirabile*, a complex structure of vessels, both presenting an abundant number of adipocytes, were used as positive controls, when treated with the different techniques; and as negative controls, when non-treated by the different techniques. Sperm whales´ lungs negative to fat emboli were also used as negative controls.

All the work in the laboratory was performed following strictly the handling and protection measures detailed in the different reagents’ safety data sheets.

### OsO_4_ technique

OsO_4_ crystals from one-gram commercial ampoule were carefully introduced in distilled water. Distilled water was pipetted several times inside the ampoule to take out all the crystals. The solution was then sonicated in periods of 10 minutes with 2 hours break until all the crystals dissolved. When sonicating, ice brick block freezers were placed in contact with the bottle containing the solution to prevent the solution from heating. During the breaks, the solution was kept hermetically sealed in the fridge at 4 °C. Afterwards, the protocol developed by Abramowsky and colleagues (1981) was followed to fix/stain with OsO_4_ to demonstrate fat in paraffin sections^18,42^. Hematoxylin and eosin were used for counterstaining.

### Chromic acid technique

In this case, chromic acid was used to fix the lipids to the tissues before paraffin-embedding. For that, the protocol described by Tracy and Walia (2002) was followed in detail^4^. As chromic acid fixes but does not stain lipids, lung sections were later stained with Oil O Red and counterstained with Mayer´s hematoxylin for fat demonstration.

For the Oil Red O staining technique, various organic solvents, as propylene glycol or isopropyl alcohol, have been proven to successfully dissolve Oil Red O to stain fats^44^. In the present study both solvents were tested, and finally, isopropyl alcohol was selected. This solvent allowed the proper staining of fat emboli since propylene glycol caused artifacts, i.e. Oil Red O clumps, hindering the proper evaluation of the tissue.

### Frozen section technique

Formalin-fixed lung sections were rinsed in phosphate-buffered saline (PBS, pH = 7.4) for two days, and then cryoprotected in 30% sucrose solution in PBS (pH 7.4) at 4 °C to avoid freezing artifacts until lung samples sank. Then lung sections were cut with a cryostat and placed in Vectabond-treated glass slides, to ensure a proper adherence of the lung sections. To demonstrate the lipids, slides were stained with Oil Red O, dissolved in isopropyl alcohol, and counterstained with Mayer’s hematoxylin.

### Microscopic analysis

We evaluated the area occupied by fat emboli in lung sections treated for the different techniques using light microscopy (Olympus BX51).

For this purpose, each lung tissue section was divided in “N” number of 100× magnified microscopic fields (MF) (Ocular: 10× and Objective: 10×) (100 MF = 1,920,000 pixels). Then, a Bootstrap analysis was carried out to determine the number of 100 MFs (“n”) that needed to be considered for each tissue section. Depending on the total lung tissue section area the Bootstrap analysis yielded results between 14 and 20 100 MFs, that were randomly selected and photographed^42^. As a result, for each animal we ended up having an “n” number of 100 MFs (photographs), each of them with an area, in pixels, occupied by fat emboli. Fat emboli were recognized as black/red droplets primarily in the lumen of capillaries and small- and medium-sized arteries. The area occupied by fat emboli in each of the 100 MFs’ fields studied was determined manually in number of pixels using ImageJ software (1.48 v, Wayne Rasband, National Institute of Health, USA). Manual selection was carried out as not all the emboli were homogeneously stained and not all the black, in the osmium tetroxide technique; or red areas, in the frozen and chromic acid techniques, were fat emboli.

### Statistical analysis

To compare the capacity of fat emboli detection of the three techniques, the proportion of fat emboli in the lung section studied was calculated for each techniquefollowing the equation:

$$FE\,proportion=a/(n\times A)$$

Where $a$ was the total pixels occupied by fat emboli in the lung section studied, calculated as the sum of the pixels occupied by fat emboli in the ‘n’ 100 MFs´ fields studied, $n$ was the number of 100MFs studied and A was the area occupied by each 100MFs (=1,920,000 pixels).

The Wilcoxon signed-rank test (a non-parametric test for two related samples) was used to assess if the mean rank between the OsO_4_ and the chromic acid, on one hand; and the OsO_4_ and the frozen section techniques, on the other hand, differ. Statistical significance was set at p < 0.05. Data were analyzed using the R package, version 3.3.1^45^.

### Evidence of ethical approval

Required permission for the management of stranded cetaceans was issued by the environmental department of the Canary Islands’ Government and the Spanish Ministry of Environment. No experiments were performed on live animals.

## Results

**Figure 1:**
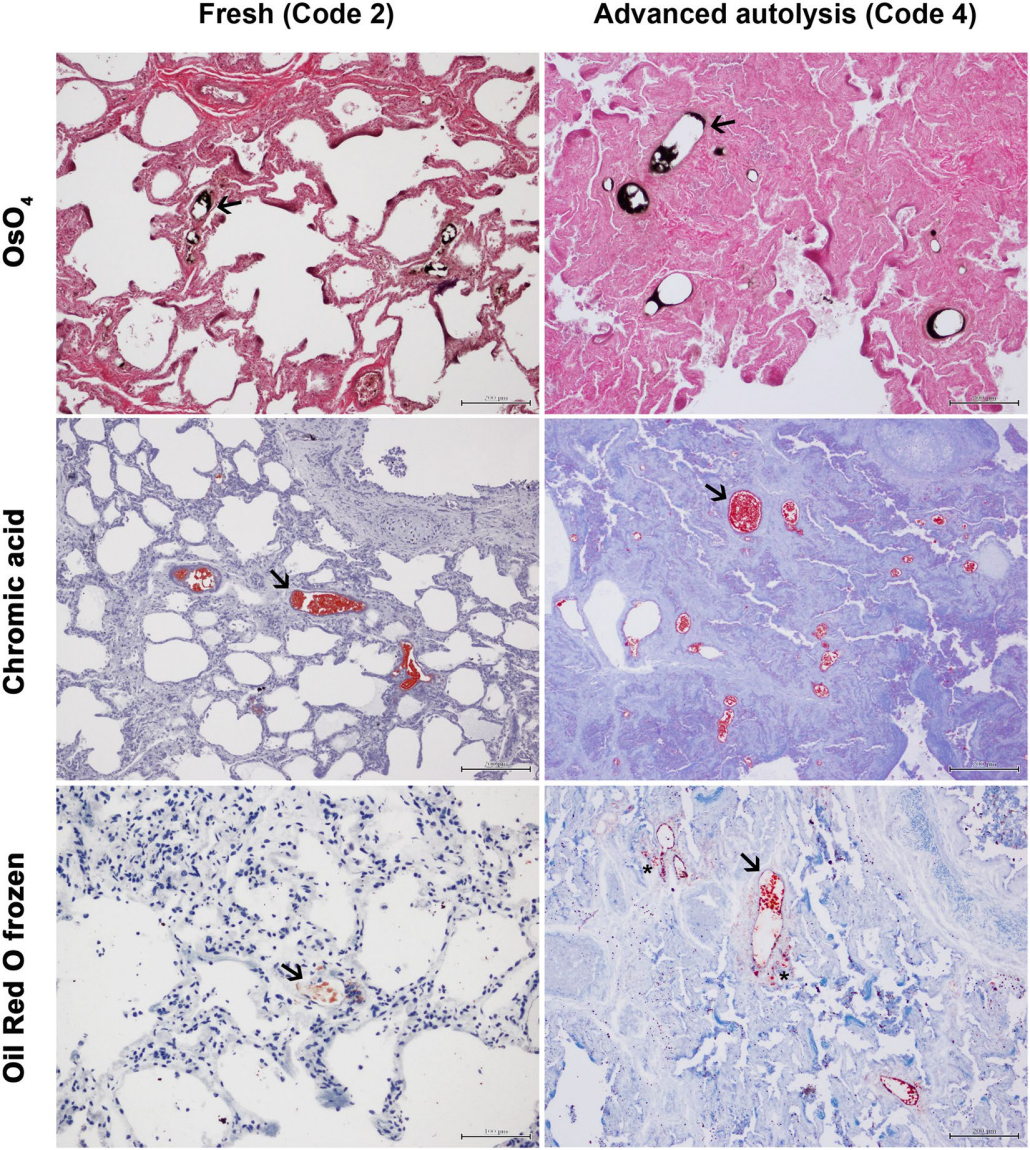
Comparison of the OsO_4_, chromic acid and Oil Red O frozen-section techniques to detect fat emboli in the lung. Left images correspond to a fresh sperm whale and right images to an advance decomposed sperm whale. Examples of fat emboli are pointed out with arrows. Asterisks in the right-bottom image point to the surpassing vessel’s limit. Bars = 200 µm, except in the left-bottom image where Bar = 100 µm.

Among the eight animals studied, 75% presented an advanced (n = 5) or very advanced (n = 1) autolysis, and two cases were fresh or presented moderate autolysis. The three techniques were able to detect fat emboli in the lungs of the animals studied, even in carcasses presenting an advanced autolysis (Fig. 1). These emboli were mainly present in medium and small caliber intrapulmonary arteries and/or obliterating arterioles.

In the OsO_4_ technique, the background presented a light to dark pink coloration, erythrocytes were very apparent in bright pink and cellular nuclei in purple, while fat emboli were stained in black. In the chromic acid and frozen section techniques, lipids appeared in red while the background (including erythrocytes and cellular nuclei) presented different shades from blue to purple, in the chromic acid technique; and from light blue (background) to dark blue (capillaries and nuclei) in the frozen section technique. The three techniques kept the tissue morphology although tissue components were more easily recognized at lower magnifications in the OsO_4_ technique, as they stained in different colors, being the chromic acid, the technique showing the least differentiation (Fig. 1).

When referring to the fat emboli, in the OsO_4_ and chromic acid techniques, although the center of the emboli was usually lost, the emboli borders appeared well fixed and stained. The center of the emboli was best preserved in the chromic acid technique. In the frozen section technique, the fat emboli detected were of lower quality, generally presenting diffuse staining and surpassing vessels’ limits (Fig. 1).

No significant differences in the fat emboli proportion could be detected when comparing the OsO_4_ technique to both: the chromic acid (p = 0.4609) and the frozen section (p = 0.7998) techniques.

## Discussion

Fat embolism constitutes a pathological finding that has been extensively described in association with various processes. It is a very common finding, especially after traumatic injuries, and it is usually clinically asymptomatic^46^ but can occasionally lead to a clinical disorder known as fat embolism syndrome (FES)^3,46^.

In cetaceans, as well as in other species, including humans, the technique traditionally chosen to assess histologically fat embolism has been OsO_4_. However, this technique has proven to be acutely toxic, requiring the implementation of extreme measures that should be strictly followed when working with it.

It is also important to highlight that, in the last years, health professionals (including veterinary pathologists and technicians) have been asked to follow new improved biosecurity measures in the field and laboratory diagnostic activities. Among these regulations, the implementation of histochemical techniques implying a low or no risk for the staff working in Veterinary Forensic Labs constitutes a requirement^47^. Considering this, a gradual transition to less toxic and more commonly used reagents, to minimize the risk of exposure, must be carried out.

With this objective in mind, chromic acid and Oil Red O frozen techniques were tested to assess fat embolism in ship-strike sperm whales´ lungs and compared to OsO_4_, since they are less toxic and require reagents commonly used in different laboratory protocols. Results showed that both techniques preserved properly the lung tissue morphology and were able to histologically demonstrate fat emboli in the lungs of sperm whales presenting different decomposition codes that were kept in formaldehyde for years, as OsO_4_ demonstrated in a recent study^42^.

When assessing quantitatively the capacity of each technique for fat emboli detection, no significant differences were detected when comparing the area occupied by fat emboli in the OsO_4_ to the other two techniques. However, the sample size was small and thus, the power to detect differences was low. Thus, future studies with a higher number of individuals should be carried out to enhance the statistical power. To our knowledge, this is the first study comparing quantitatively the detection capacity of the three techniques. There is just one previous study that attempted to compare OsO_4_ and Oil Red O frozen section techniques for fat emboli detection^18^. Conclusions of this study showed that tissues treated with OsO_4_ showed a higher degree of staining compared to the Oil Red O frozen section technique when small quantities of fat were present. When comparing these results to ours, it should be highlighted that: first, in the present study fat emboli were especially present in small size arteries and arterioles and not in capillaries, thus, the capacity of fat emboli detection when minute amounts of fat were present could not be compared among techniques; and secondly, while in the present study fat emboli area, followed by statistical analysis, is used for comparison between techniques, Abramowsky and colleagues followed the Mason’s 1 to 4 scale, based on the number of emboli present in the tissue^16^, which is a much less accurate methodology that scores fat emboli based on the observer personal criteria rather than in an objective quantitative grading system.

Apart from being necessary to make objective comparisons among techniques, the proper assessment of the amount/area of fat emboli present in a tissue section (fat embolism proportion) is essential as the implications of those emboli in the individual’s death may vary significantly among grades. Considering this, the present study method has not only efficiently compared the techniques studied, but it constitutes an objective grading system that can be implemented in further studies aiming to properly assess fat embolism severity.

Among the three techniques, OsO_4_ and chromic acid showed a superior fat emboli-staining quality compared to the frozen section technique, which is the least toxic. In the former ones, the intensity of the staining was superior and the emboli showed well-defined borders allocating them precisely within arteries and arterioles contrary to the third technique, in which lipids tended to move during the tissue processing as they were not previously immobilized (fixed). These differences were already noticed by Abramowsky and colleagues when comparing the OsO_4_ and the Oil Red O frozen technique^18^. Better preservation of the emboli center in the chromic acid compared to the OsO_4_ technique could be explained because the osmium only reacts with unsaturated fats, being unable to demonstrate saturated fats^48^. In the chromic acid technique, this limitation may be resolved by infiltrating the formalin-fixed tissue with an emulsion of a polyunsaturated fatty acids (linoleic acid) in ethylene glycol. These unsaturated lipids partition into the tissue lipids, enriching the number of unsaturations reacting with the chromic acid^4^. This advantage, together with its higher capacity of penetration, its more common use in laboratories, and its lower price, make chromic acid an interesting alternative to OsO_4_ for histological fat emboli detection.

Further histochemical studies on fat embolism using other microscopy techniques, like confocal microscopy, will probably benefit from the features and possibilities of modern and more complete equipment. Other techniques, such as immunohistochemistry, have been recently described for fat embolism assessment. In these cases, although fat deposits are lost in the process of paraffin-embedding, the finding of fibrinogen and platelets around those empty spaces has been proposed as a proof of intravital fat embolism^49^. However, this should be taken carefully as in cases of decompression sickness, platelet^50–52^, and less frequently fibrinogen^50^, aggregation around the gas bubble surface has been demonstrated as well. In cetaceans, gas and fat embolism have been jointly described in beaked whales stranded in association with sonar activities^38,39^, thus, techniques able to histologically demonstrate those fat emboli are essential. Considering this, immunohistochemistry analysis for fat embolism assessment should always be taken as a complementary diagnostic tool, especially useful when fresh frozen or fixed samples are not available, and not as a substitute of techniques able to visualize the fat emboli.

In conclusion, the three techniques were proven to be valuable for the diagnosis of fat embolism in cetacean tissues, even in decomposed tissues and after long periods in a fixative solution. Although quantitative differences could not be established, the chromic acid technique showed a higher quality in fat emboli staining than the Oil Red O frozen technique. The chromic acid technique also stands as a good alternative to OsO_4_ due to its equivalent capacity to detect fat emboli, or even superior when considering the preservation of the emboli center. It is also commonly used in other laboratory procedures and has minimal costs when compared with the expensive OsO_4_. Although in the present study the different techniques were tested in sperm whales, these results are of application in human and animal forensic medicine.
